# Phenol-rich fulvic acid as a water additive enhances growth, reduces stress, and stimulates the immune system of fish in aquaculture

**DOI:** 10.1038/s41598-020-80449-0

**Published:** 2021-01-08

**Authors:** Thora Lieke, Christian E. W. Steinberg, Bo Pan, Irina V. Perminova, Thomas Meinelt, Klaus Knopf, Werner Kloas

**Affiliations:** 1 Department of Ecophysiology and Aquaculture, Leibniz-Institute of Freshwater Ecology and Inland Fisheries, Berlin, 12587 Germany; 2 Faculty of Life Sciences, Humboldt University of Berlin, Berlin, 10115 Germany; 3 Faculty of Environmental Science and Engineering, Kunming University of Science and Technology, Kunming, 650500 China; 4 Lomonosov Moscow State University, Leninskie Gory, 119991 Moscow, Russia

**Keywords:** Sustainability, Innate immunity, Environmental impact, Environmental impact, Ecology, Freshwater ecology

## Abstract

Aquaculture has become imperative to cover the demands for dietary animal protein. Simultaneously, it has to overcome prejudices from excessive use of antibiotics and environmental impacts. Natural supplements are traditionally applied orally. In this study, we demonstrated another pathway: the gills. Humic substances are immunostimulants and a natural part of every aquatic ecosystem, making them ideal to be used as bath stimulants. Five and 50 mg C/L of a fulvic acid-rich humic substance was added for 28 days to the water of juvenile rainbow trout (*Oncorhynchus mykiss*). This fulvic acid is characterized by a high content of phenolic moieties with persistent free radicals and a high electron exchange capacity. The high concentration of the fulvic acid significantly increased growth and reduced the food conversion ratio and the response to a handling-stressor. Phagocytosis and potential killing activity of head kidney leukocytes were increased, as well as the total oxyradical scavenging capacity (TOSC) and lysozyme activity in the gills. In conclusion, immunostimulation via gills is possible with our fulvic acid, and the high phenolic content improved overall health and stress resistance of fish.

## Introduction

Fishes play an important role to cover the demands of animal protein, and FAO projected the aquaculture production to exceed capture for the first time in 2020^1^. Apart from the increasing demand, aquaculture faces a major problem: tremendous prejudices of consumers because of the excessive use of antibiotics and other chemicals in the past and negative impacts on the environment. Stimulating the immune system with feed additives to help prevent diseases and the use of therapeutants is becoming a far-reaching practice in fish production^2–5^. There are several downsides to this application method: Firstly, complementing the feed requires additional processing, which increases the costs. Secondly, the concentration inside the fee is fixed and cannot be adjusted easily by the fish farmer depending on the current requirements. And thirdly, because of feed competition, not all fish inside the same batch ingest the same amount of additive, making the effects unpredictable (too low concentrations can have no effects, too high can have adverse effects). In fish, there is a second possible route of immunostimulant uptake: the gills. The large surface area of gills is not only necessary to guarantee sufficient oxygen uptake but is an entrance portal for microorganisms and xenobiotics^6,7^. Furthermore, the gill associated lymphoid tissue^7^ plays an important role in the immune response and would be in direct contact with the bath stimulants. Nevertheless, research on stimulating the immune system via water treatment is still scarce^8,9^. A long-term application, similar to the use of immunostimulants in feed, has not been studied yet. Possible reasons for those lacks are that bath stimulants have to cover several requests: complete water-solubility (excluding high molecular molecules such as ß-glucans from algae and fungi or chitin), non-irritant (excluding many herb extracts), and, due to the required large amounts, they have to be cheap to be economically realistic (excluding many vitamins).

Humic substances (HS) are part of natural organic matter and represent up to 95% of dissolved organic matter (DOC) in aquatic ecosystems with concentrations normally ranging from 0.5 mg C/L to 50 mg C/L^10–12^. They are “complex and heterogeneous mixtures”^13^ and their structure can differ greatly depending on their origin: Comparing 20 HS Meinelt, et al.^14^ found not only enormous differences between different lakes but also seasonal changes in the structure of HS within one lake. This chemical diversity is reflected by the heterogeneity of biological effects.

Beneficial effects of HS, when used as feed additives, include growth stimulation, reduced mortality, accelerated recovery after diseases, and stimulation of immune-related genes^15–18^. However, negative or contradictory effects are reported as well, including genotoxicity in blood lymphocytes, oxidative stress, and reduced offspring in *Daphnia magna*^19–21^. Only a few quantitative structure–activity relationships of humic substances have been conducted so far, but they showed that aromatic moieties and carboxyl/esterified functions have opposite effects in maize and *Saprolegnia parasitica*^14,22^. Although not directly transferable to fish, these results showed that the structure of the HS plays a tremendous role in its biological effects. To compare the effects of different humic substances, the chemical characterization cannot be neglected.

Humic substances often occur as waste-products during drinking water production and because of their aquatic origin, they are ideal to be used as water additives. The present study aimed to close knowledge gaps on two main aspects: Firstly, can humic substances, applied as bath treatment, stimulate the immune system of fish, and secondly, which chemical structures are characteristic for the humic substance applied, being potentially responsible for the biological effects.

## Results

### FulvoFeed (FF) is a small molecular fulvic acid with high phenolic moieties and EDC

The liquid FF has a dry content of 242.93 g/L and a carbon content of 97 g/L. As the analysis of elemental composition requires previous dry freezing, Table 1 shows directly measured results of dry content (mg/L) and calculated content of liquid FF(g/L).

**Table 1:** The elemental compositions of FF particles and solution.

	C	O	N	H	S
Dry FF (mg/g)	398.1	537.1	6.5	50.0	8.3
Liquid FF (g/L)	96.7	130.5	1.6	12.2	2.0

As natural products, humic substances are a mixture of different carbon-based structures. Liquid Chromatography-Organic Carbon Detection-Organic Nitrogen Detection (LC-OCD-OND) analysis showed, that the carbon-structures consists of 0.1% bio-polymer, 4.2% building blocks (low molecular structural elements of HS), 7.6% low molecular weight substances, and 88.2% humic-like substances with an average molecular weight of 800 g/mol. Of these humics, 94.4% are fulvic acids and 5.6% are humic acids. As measured by^13^C-NMR spectroscopy, the amount of different structural groups was as follows: 29.2% aromatic carbon (including 7.6% phenolic carbon), 20.3% carboxyl carbon, 5.4% carbonyl carbon, 12.7% carbohydrate carbon, and 28%—non-substituted aliphatic carbon. Electron donor capacity (EDC) was 380 μmol/g and electron acceptor capacity (EAC) was 270 μmol/g. Electron paramagnetic resonance, as a measure of persistent free radicals, reveals a g-factor of 2.00453 and an intensity of 4.6*10^6^/g carbon.

### Bath treatment increases growth and reduces feed conversion ratio (FCR)

**Figure 1:**
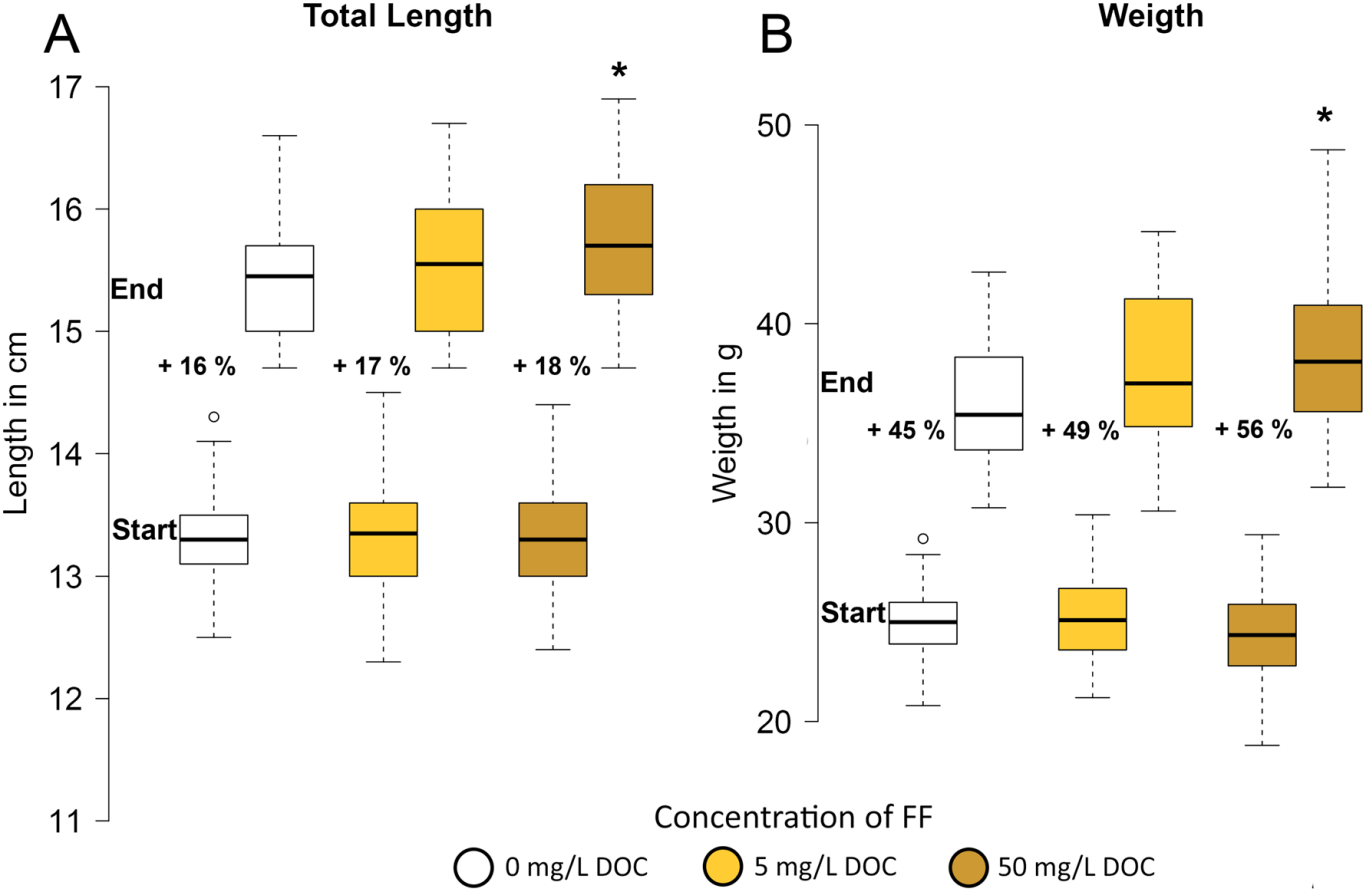
Mean total length **(A)** and mean weight **(B)** of juvenile rainbow trout at start and end (28 days) of exposure to FF. Percentages represent gains relative to the start value. *p < 0.05 against control.

Juvenile rainbow trout had a mean length of 13.3 ± 0.4 cm and a mean weight of 24.9 ± 2.0 g when starting the experiment. After 4 weeks, control fish had gained a length of 15.4 ± 0.4 cm (16.0% increase) and a weight of 36.2 ± 3.1 g (45.0% increase) (Fig. 1A,B). Exposure to FF led to increased growth: fish exposed to 5 mg C/L had a total length of 15.5 ± 0.52 cm (16.5% increase) and a weight of 37.6 ± 3.65 (48.8% increase), while fish exposed to the high concentration (50 mg C/L) gained significantly more length and weight (18.2% length and 56.4% weight) resulting in a total length of 15.7 ± 0.6 cm and a weight of 38.2 ± 4.0 g. There was no mortality.

At the same time, the mean absolute growth rate (AGR), was increased, from 0.401 ± 0.01 in control fish to 0.440 ± 0.05 in fish exposed to the low concentration of FF and significantly to 0.493 ± 0.05 (p < 0.05) in fish exposed to the high concentration of FF. Furthermore, the mean feed conversion ratio (FCR) was reduced with a trend visible in the 5 mg C/L and a significant decrease (p < 0.05) in the 50 mg C/L group (Control: 1.24 ± 0.03; 5 mg C/L: 1.13 ± 0.15, 50 mg C/L: 1.00 ± 0.10). There was neither a difference in the K factor (1.4 ± 0.1) nor the hepatosomatic index (HSI) (9.0 ± 1.9), the spleen somatic index (SSI) (0.9 ± 0.3) or the weight ratio of visceral fat to total weight (3.7 ± 2.0 mg/g) or visceral fat to organ weight (0.05 ± 0.02 mg/mg).

### FulvoFeeds increases stress resistance

**Figure 2:**
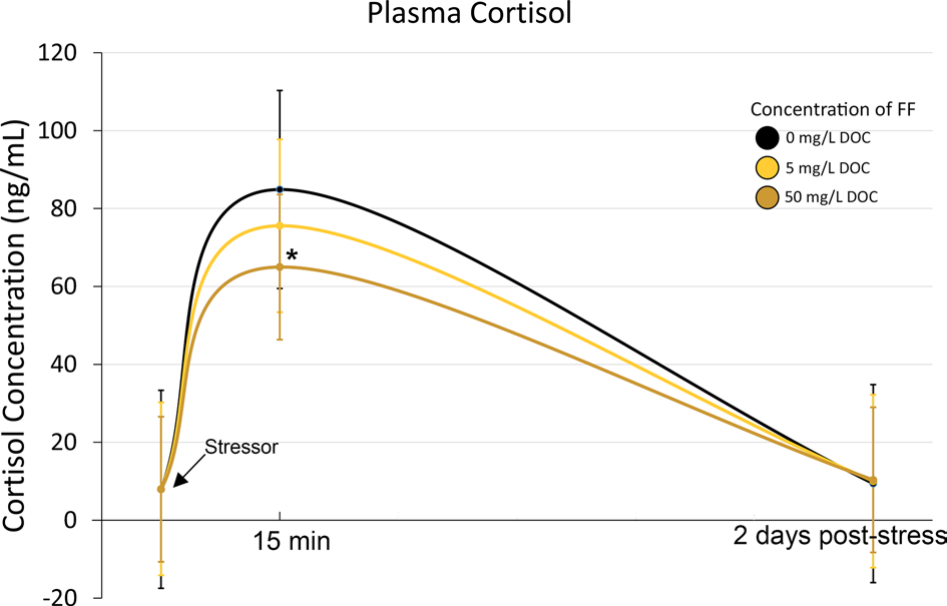
Plasma cortisol concentration after 28 days of exposure, 15 min post stressor (netting and 30 s air-exposure), and 2 days post-stress. N = 12, mean ± SD; *p < 0.05 against control.

After 4 weeks of exposure, the plasma cortisol concentration was 8 ± 3 ng/mL showing no difference between the three groups. Fifteen minutes after netting and air-exposure, cortisol concentration in control fish increased approximately eleven times compared to the baseline value (85 ± 19 ng/mL), while that of fish exposed to 50 mg C/L FF increased only eight times (65 ± 13 ng/mL) (Fig. 2). The trend to a lower cortisol concentration in response to stress was also detectable in fish exposed to 5 mg C/L of FF (76 ± 21 ng/mL). Plasma cortisol concentration in all fish at the 2nd-day post-stress was back to the baseline concentration without differences between the groups.

### Exposure stimulates innate defense mechanisms in the head kidney but not in plasma

**Figure 3:**
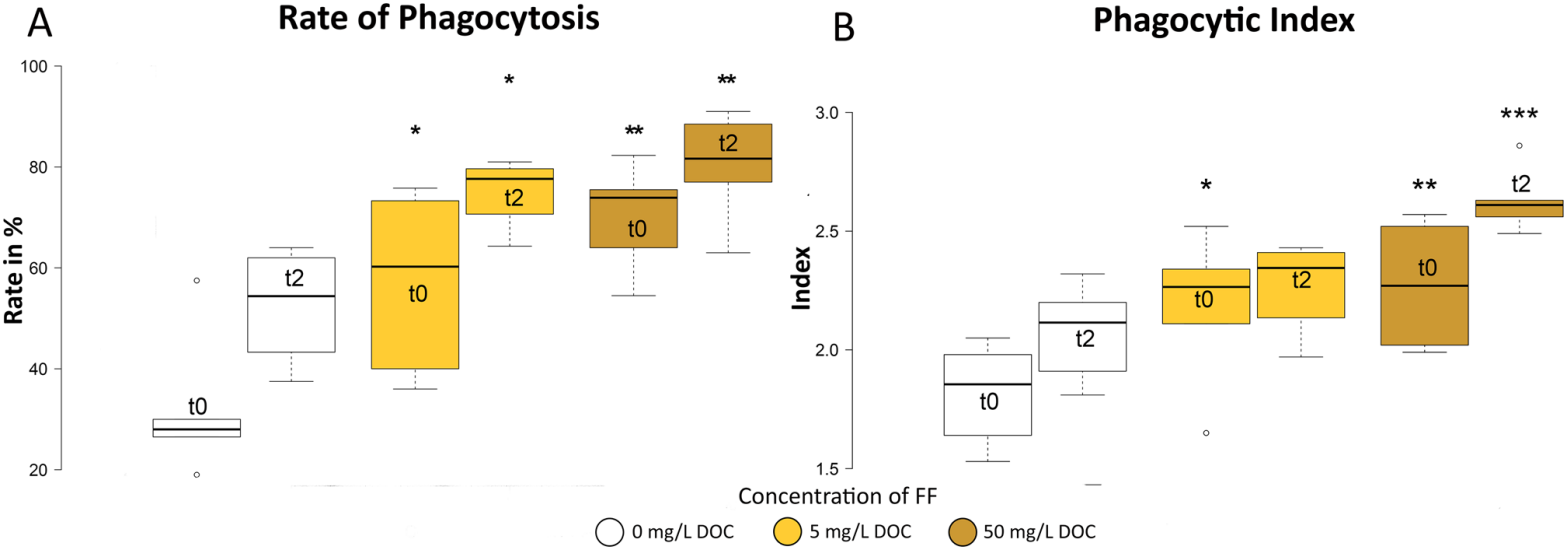
Rate of phagocytosis **(A)** and phagocytic index **(B)** of head kidney cells. t0: 28 days of exposure; t2: 2 days post stressor (t0). *p < 0.05; **p < 0.01; ***p < 0.001. n = 6.

Phagocytes isolated from the head kidney of fish treated with both concentrations of FF had a significantly increased rate of phagocytosis and phagocytic index (Fig. 3). While the cells from control fish had a rate of phagocytosis of 31.5 ± 13.3% and a phagocytic index of 1.8 ± 0.2, cells from fish exposed to the low concentration had a rate of 57.6 ± 17.3% and an index of 2.2. ± 0.3. In cells from fish exposed to the high concentration, the rate increased to 70.7 ± 9.9% and the index to 2.3 ± 0.3. Two days after the stressor, the rate in control fish increased to 52.6 ± 10.5% and the phagocytic index to 2.1 ± 0.2. Again, cells from FF exposed fish showed a significantly higher rate of phagocytosis (75.2 ± 7.4% in the low exposure group and 80.5 ± 10.0% in the high exposure group) and phagocytic index (2.3 ± 0.2 and 2.6 ± 0.1, respectively).

**Figure 4:**
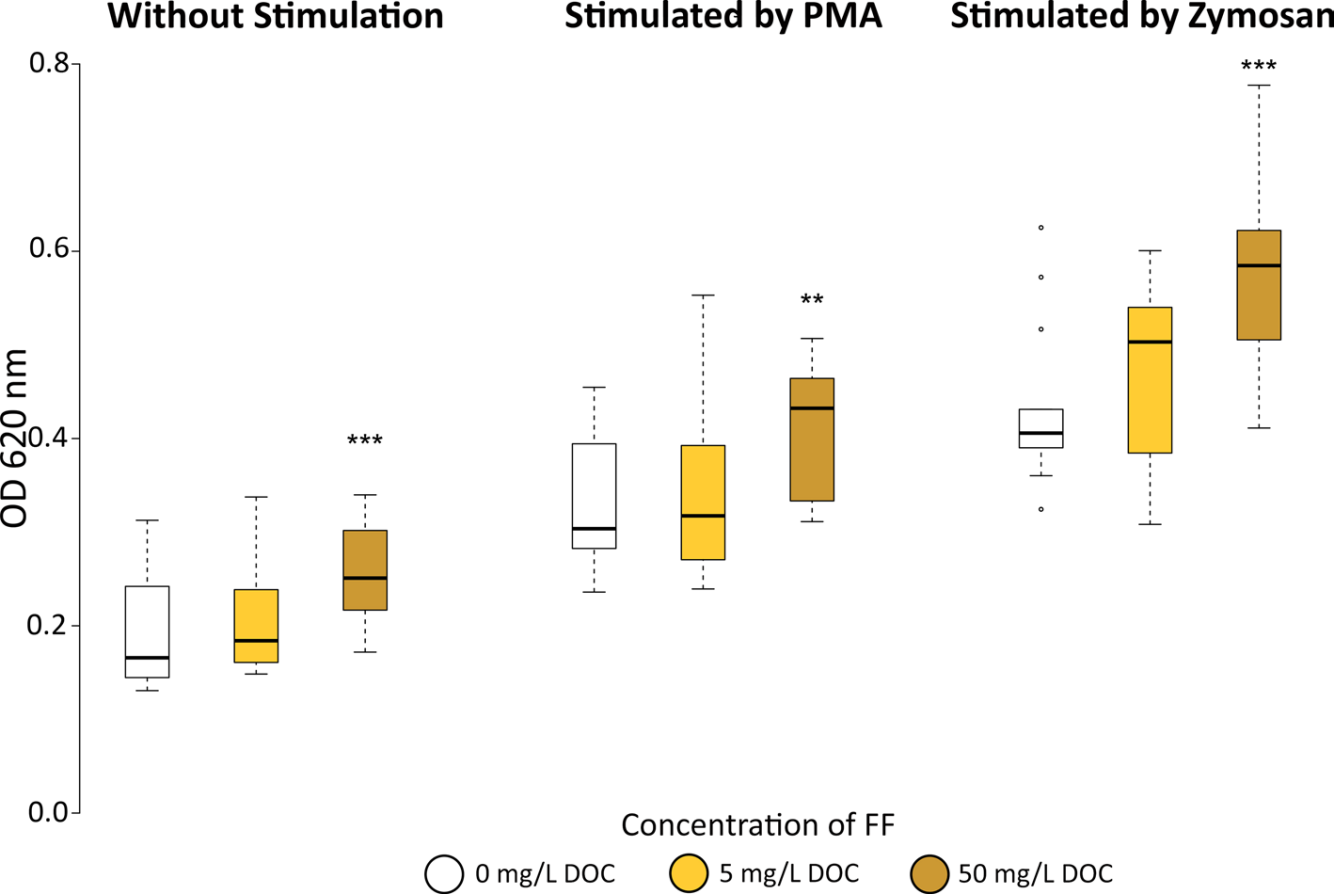
Production of reactive oxygen species of head kidney leucocytes measured by NBT (nitro blue tetrazolium) assay. **(A)** Without additional stimulation; **(B)** using PMA (phorbol 12-myristate 13-acetate) as a stimulant; **(C)** using zymosan as a stimulant. Optical density (OD) was measured at 620 nm. **p < 0.01; ***p < 0.001. n = 6.

Before stressing, the head kidney cells from fish exposed to the high concentration of FF produced significantly higher amounts of reactive oxygen species (ROS) (OD (optical density) 620: 0.26 ± 0.05) compared to the cells from control fish (OD 620: 0.19 ± 0.06) (Fig. 4). This trend was also visible in the cells from fish exposed to 5 mg C/L FF (OD620: 0.21 ± 0.07). After stimulation with phorbol 12-myristate 13-acetate (PMA), the ROS production in all groups increased by 68 ± 20%, while zymosan increased the production by 145 ± 40%. Again, the cells from the high FF concentration group showed significantly higher respiratory burst activity as compared to the control (OD 620: 0.42 ± 0.07 with PMA and OD 620: 0.58 ± 0.1 with zymosan). Cells from fish exposed to 5 mg C/L showed intermediate responses (OD 620: 0.35 ± 0.09 with PMA and OD 620: 0.47 ± 0.09 with zymosan). Two days post-stressor, there was no significant difference between the groups; neither before (OD 620: 0.14 ± 0.06) nor after induction of the respiratory burst (OD 620: 0.24 ± 0.10 with PMA, 0.40 ± 0.16 with zymosan).

The protein content of plasma in control fish was 22.3 ± 1.6 mg/mL and was not altered by exposure to FF. The same applies to the albumin: immunoglobulin ratio (1.9 ± 0.3) that was not affected by exposure. Furthermore, lysozyme activity of plasma was not changed by exposure (control: 44.0 ± 6.9 U/mg protein; 5 mg C/L: 42.1 ± 8.3; 50 mg C/L: 45.7 ± 7.0 U/mg protein).

### Defense mechanisms of gills are stimulated by exposure to FF

**Figure 5:**
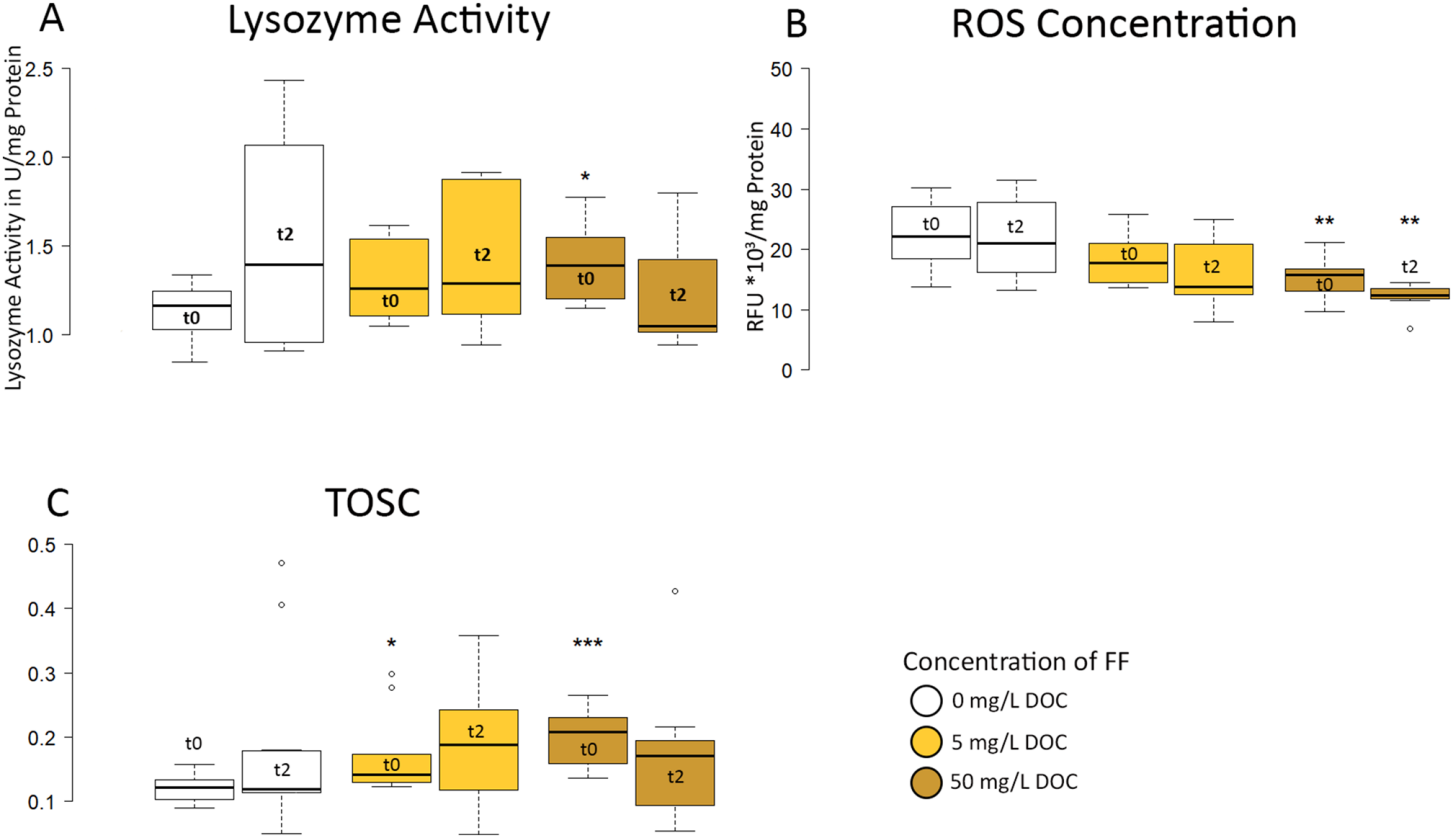
Defense mechanisms in gill supernatant. **(A)** Lysozyme activity, **(B)** concentration of reactive oxygen species (ROS), **(C)** total oxyradical scavenging capacity (TOSC). t0-28 days of exposure; t2-2 days post-stressor (t0). *p < 0.05. **p < 0.01; ***p < 0.001. n = 12.

The protein content of gill extracts (4.0 ± 0.3 mg/mL) was not altered by exposure to FF and used as normalization for lysozyme activity. The lysozyme activity of fish exposed to 50 mg C/L FF was significantly increased at the first sampling time point (Fig. 5A). The activity of control gills was 1.1 ± 0.2 U/mg protein, while fish from the high exposure group had an activity of 1.4 ± 0.2 U/mg protein. A trend towards an increased activity was detected in the low concentration group (1.3 ± 0.2). Two days after the stressor, the activity increased in all groups to an average of 1.4 ± 0.5 U/mg protein, without significant differences between the groups.

The concentration of ROS in gills decreased significantly in the fish exposed to high concentrations of FF as compared to the control group (Fig. 5B; Control: 22.4 ± 5.6, 5 mg C/L: 18.4 ± 4.0, 50 mg C/L: 15.9 ± 3.4). This applies to both sampling time-points: after 28 days of exposure and 2 days after the first sampling (and the stressor). A trend towards a reduced ROS concentration was also visible in the group exposed to the low concentration of FF. At the same time, the total oxyradical scavenging capacity (TOSC) of fish after 28 days of exposure was significantly increased in both groups compared to the control (Fig. 5C, Control: 0.12 ± 0.02, 5 mg C/L: 0.17 ± 0.06, 50 mg C/L: 0.21 ± 0.04). Two days post stressor, there was no difference in the TOSC activity between the groups.

## Discussion

### Biological effects of humic substance have to be referred to chemical properties

FulvoFeed has an average molecular mass of 800 g/mol and contains primarily fulvic acids and a small amount of humic acids. Relatively small molecular masses (< 3.5 kDa) can interact with plasma membranes, causing high biological reactivity^23–25^. This applies not only to plants but to aquatic animals as well. Fulvic acids are the low molecular weight fraction of humic substances and, therefore, their uptake over the gill epithelial surface or implementation into the mucus -or both- is highly possible.

The aromatic content of FF is around 30%, which is in the higher range of aquatic fulvic acids. At the same time, its aromatic core is heavily substituted with phenolic groups which constitute 26% of the total aromatic carbon (Perdue^26^; Table S1). The EPR spectrum indicates a high content of persistent free radicals (PFRs). This can be attributed to the high phenolic content and confirms the results from our NMR spectrum^27^. In previous studies, we showed that PFRs exert stimulation to several parameters including the growth of plants and neuro-behavior of *Caenorhabditis elegans* when applied at low concentrations and become toxic at higher concentrations^28,29^. Furthermore, FF has a high EDC (380 µmol/g), compared to DOC from compost (< 100 µmol/g) and a high EAC (270 µmol/g) compared to Suwannee River standard fulvic acid (< 100 µmol). Both result in a high electron exchange capacity (EEC), reflecting its high ability to act as an electron shuttle^30,31^.

### Increased resistance does not cause the growth stimulation

**Figure 6:**
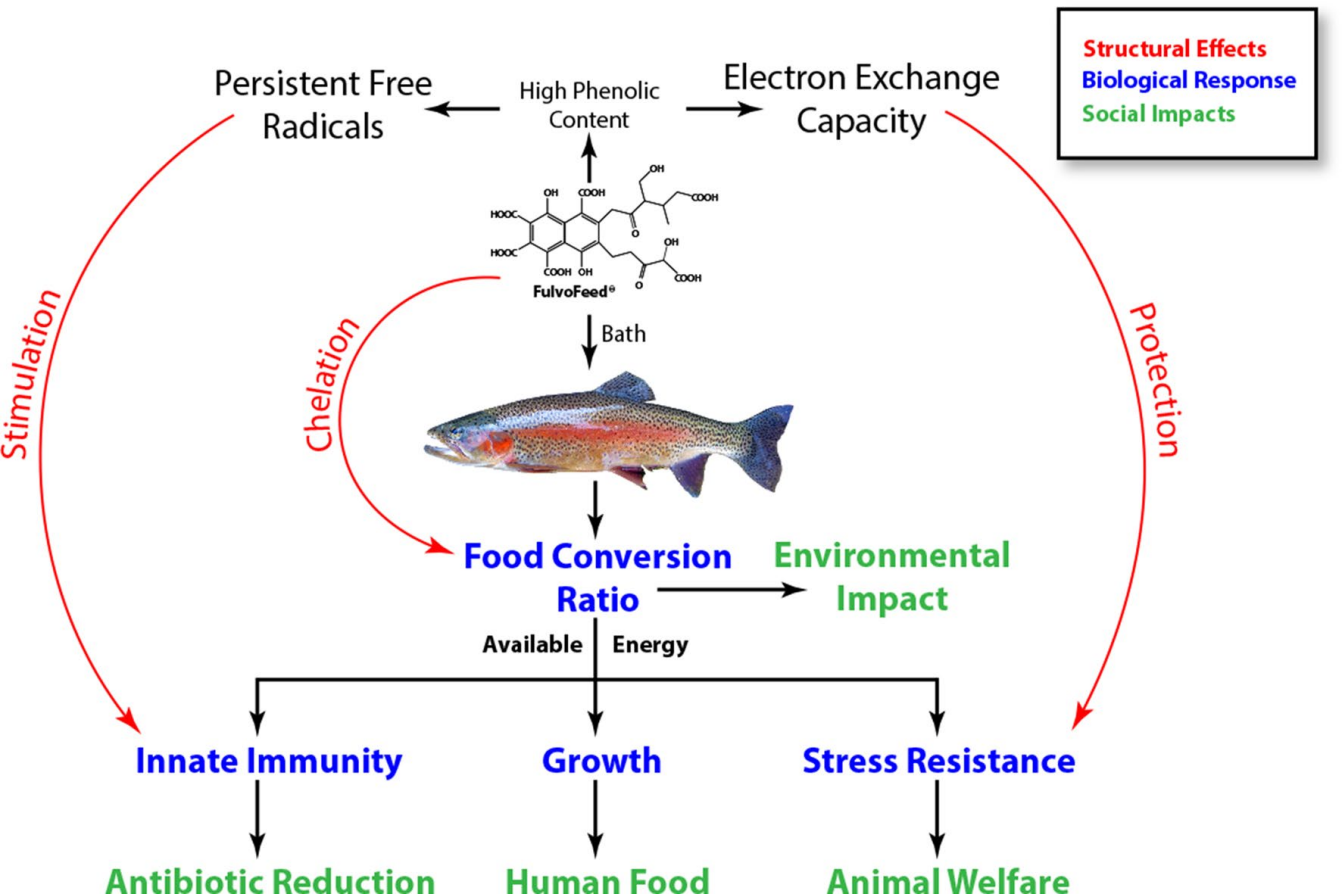
Overview of results from this study.

By adding FF for 4 weeks to the rearing water of juvenile rainbow trout, we were able to increase length and weight gain by 5% and 10%, respectively, and to significantly improve feed conversion by around 16% in fish exposed to the high FF concentration. These results are comparable to those obtained in another study using immunostimulants as feed additives^32^. Against the background of the involvement of beneficial gut-bacteria in growth stimulation, it is intriguing that water supplementation with FF resulted in stimulated growth parameters as well, as there is little direct contact between the gut and the substance. As mentioned, uptake of FF and further distribution via blood is possible. The significant reduction of FCR indicates a better digestion efficiency and utilization of feed. Gao, et al.^18^ reported increased intestinal digestive enzyme activity (protease, lipase, and amylase) on feed supplementation with fulvic acid, and chelation of mineral ions might promote the nutrient uptake and utilization of minerals in feed^33^. Eventually, resulting in an increased amount of energy gained from the feed, which can then be allocated for maintenance of homeostasis, growth, and defense mechanisms. An improvement of the FCR furthermore reduces the nutrient output (especially nitrogen and phosphorous) and the production of greenhouse gases, decreasing the impact on water bodies (if open systems are used) or wastewater production (in closed aquaculture recirculating systems) and the carbon footprint of aquaculture^34,35^. Figure 6 shows a graphical summary of our results.

Stress, especially chronic one, and the allostatic load imposed to cope with it, significantly affects the energy metabolism and enhances the production of extra energy supply from body resources^36,37^. At the same time, stress leads to reduced or arrested feed intake and depressed immune system functions, impairing growth performance, and leaving the fish vulnerable to infections^38–40^. Cortisol is most widely used to define stress levels^41^. Exposure to FF did not affect the cortisol concentration in blood, indicating, that it did not exert any stress on the fish. In the laboratory, fish were kept under ideal conditions and external stress was kept at a minimum; it is therefore unlikely, that a reduced stress during the exposure time accounts for the improvement detected in growth and FCR.

To mimic inevitable handling in aquaculture, we exposed fish to a strong acute stressor (netting and 30 s of air exposure). The cortisol response of fish, conditioned with 50 mg C/L FF was 24% lower than that of unexposed fish. It can be reasonably assumed, that other stressors will be diminished as well. Further analyses, especially on molecular levels, are needed to work out the modes of action, but we were able to show that FF addition to the rearing water helps to increase the growth of juvenile rainbow trout and reduce cortisol response to an acute stressor.

### No apparent oxidative stress was exerted to the gills

As ROSs are naturally formed by aerobic metabolism in organisms and are part of the potential killing activity of macrophages, cells have antioxidant defense mechanisms (measured as TOSC) to scavenge ROS and to maintain the cellular redox homeostasis. External oxidative stress can deplete this defense mechanism and evoke severe cellular damage. The electron paramagnetic resonance (EPR) spectroscopy showed a rather high concentration of unpaired spins in FF. However, the amount of ROS detected in the gill tissue after exposure to the high concentration of FF was significantly reduced, and the same trend was also visible in the low exposure group. Furthermore, exposure to both concentrations of FF significantly increased the TOSC of gill tissue. Together, this indicates that the applied FF did not exert an apparent oxidative stress on the gills; the overall effect is protective.

At first glance, this appears to contradict previous studies with freshwater amphipods *Gammarus sp.* and *D. magna* exposed to humic substances, where several endpoint markers of oxidative stress increased^19,42^. However, invertebrates are a lot more sensitive to xenobiotics than vertebrates^43^ and the gills of fish are covered by mucus decreasing direct contact with FF. Especially in the described case with *D. magna*, the concentrations of humic substances used were similar to the highest exposure used in our study on fish but it might be too high for potential protective effects in those invertebrates.

Furthermore, Timofeyev, et al.^42^ observed a 2-stage-response in gammarids with increased activity of antioxidative enzymes in the first stage, and cell damage, probably due to depletion of the TOSC, in the second phase. The increased TOSC after 28 days of exposure to the FF in our study indicates that depletion to cope with the overproduction of ROS generated by oxidative stress is highly unlikely. The high aromatic content and EEC might add to the TOSC by inactivating radicals. Similar effects were observed after exposing the stonewort *Chara hispida* to humic substances^23^.

Lastly, the effects found with one humic substance cannot necessarily be transferred to another humic substance. Both, the concentrations of FF and the exposure duration used in our study, did not evoke oxidative stress in rainbow trout as previously reported in invertebrates. However, whether or not the negative effects will appear at higher concentrations and/or longer exposure time has to be tested before any other long-term application can be recommended.

### Fish are prepared to deal with pathogens

Phagocytes are an important part of the innate defense mechanisms and can reasonably be expected to increase the capacity to neutralize invasive microbes^44^. Exposure to FF increased the number of active phagocytes in the head kidney by 83 and 124%, respectively. Furthermore, the number of particles digested by each phagocyte increased by 22 and 28%, respectively, and the potential killing activity increased significantly. Altogether, this increases the microbiocidal capacity^45^ allowing the fish to defend themselves against pathogens. Similar effects were determined when using an oral application of immunostimulants^46^. As chronic stress has been found to decrease the efficiency of macrophages (phagocytosis rate and ROS production)^47–49^, the FF protective function is working in two ways simultaneously: by increasing stress resistance and by stimulating the phagocytic activity and the potential killing activity of leucocytes.

Lysozyme activity was significantly increased in the gills after exposure to the high concentration of FF compared to the control, indicating an improved protection against bacterial invasion. Two days post-stressing the lysozyme activity of all groups was increased due to acute stress-induced enhancement^36,50^, however, without any difference between the groups. Unexpectedly, the plasma lysozyme activity was not affected by FF exposure. This contradicts findings from oral administration of immunostimulants including HS^18,51,52^. However, none of these studies evaluated the effects on gill lysozyme activity.

In addition to these direct anti-bacterial properties (and thereby anti-inflammatory properties), lysozyme is involved in pro-inflammatory responses and in resolving inflammation. This is why the temporal and spatial balance of lysozyme activity is crucial after the invasion of pathogens as explained by Ragland and Criss^53^.

Although lysozyme activity was increased, we did not detect an increase in the ROS concentration in the gill tissue after exposure to the FF sample, which could indicate chemotaxis and similar activation of leucocytes as in the head kidneys. There are several possible modes of action to explain this: firstly, some humic substances have antibacterial properties, reducing the overall bacterial load exerted on the gills^18,54^ reducing the need to activate the fish defenses. Secondly, increased lysozyme activity and degradation of water-borne bacteria might be “defense enough”, and, therefore, the recruitment of neutrophils and macrophages might not occur. Thirdly, the measurement of ROS concentration in the gill extract is only an indirect method to detect the potential killing activity of leucocytes. As TOSC was increased in gill tissues, ROS produced by leucocytes might have been scavenged. To determine, whether or not FF stimulates recruitment and activates gill leucocytes, further analyses including isolation of gill leucocytes and preparations of cell cultures are required. Determining transcriptional changes of pro-inflammatory signal molecules and amounts of leucocyte marker molecules can help to deepen the understanding of how FF modulates the lysozyme-mediated immune response, and whether or not pro-inflammatory effects are exerted.

Our study shows that defense mechanisms are activated by bath exposure to FF, which could help the fish to protect themselves against diseases. However, more research including challenge experiments is needed to evaluate if exposure to FF helps to reduce the susceptibility of fish against pathogens.

## Conclusion

Consumers’ interest in safe and sustainable food sources is rising. This concerns especially the use of antibiotics and harmful therapeutants, but the overall-impacts of agri- and aquaculture on resources and environment as well. Natural immunostimulants can help to increase growth and animal welfare while protecting from diseases by activating the host immune system. They are commonly applied orally; we showed that stimulation over the gills is another pathway for fish in aquaculture. Fulvic acids are part of aquatic ecosystems and bath application is a natural way to apply these immunostimulants. However, because of the heterogenic character of this substance group, chemical structures have to be determined before the biological application.

We demonstrated that the addition of FF to the water increased the growth of fish without adversely affecting the weight/ fat ratio and improved the FCR. Furthermore, the stress response was significantly lower in fish conditioned with FF after netting and air-exposure. Gills, which are the entrance portal of viral and bacterial pathogens, had significantly improved defenses in regards to lysozyme activity and protection against oxidative stress as compared to non-treated fish. The innate immunity was also significantly improved in terms of the increased activity and efficiency of leucocytes in the head kidney. The high aromatic contents of FF, especially the phenolic moieties, lead to a high EEC and protect against oxidative stress. At the same time, the PFRs in FF exert mild stress, which stimulates the immune system. Although the protection against a pathogen has to be determined yet, FF could help to reduce the use of chemical therapeutants and prevent fish from diseases. Overall, implementing FF to the water of fish is an easy and natural way to improve fish health and growth, and to decrease the impact of aquaculture on the environment.

## Material and methods

### Humic substance and chemical analysis

FF is a preparation of humic material that was obtained from HuminTech GmbH, Grevenbroich, Germany. It was extracted from groundwater in wetland/bog rich regions in northern parts of the Netherlands. Precipitation of FF was not observed in the concentrations used. Contents of carbon, nitrogen, hydrogen, and sulfur were determined by high-temperature combustion using a Vario MICRO cube (Elementar Analysensystem, Langenselbold, Germany). Oxygen content was calculated as the difference between the sum of elemental content (in mg/g) and total dry content (after freeze-drying the liquid FF). The quantitative solution-state^13^C NMR spectroscopy was performed using a Bruker DMS 400 NMR spectrometer operating at 100 MHz^13^C frequency, using 0.3 M NaOD/D_2_O as a solvent. Spectral assignments were made according to Hertkorn, et al.^55^. LC-OCD-OND was performed by DOC-Labor Dr. Huber, Karlsruhe, Germany. Mediated electrochemical reduction (MER) and oxidation (MEO) were performed at applied potentials of -0.70 V and + 0.4 V (vs. Ag/AgCl reference electrode), respectively, to measure the EEC (including , EAC and EDC)^56^. EPR spectrum was recorded on a Bruker X-band A300-6/1 EPR spectrometer (Bruker, Billerica, Massachusetts, USA) by loading liquid FF into a microcapillary^28,29,57^. Intensity and g-factor were calculated using the Bruker WinEPR Acquisition software 4.40 Rev.11.

### Experimental setup

**Figure 7:**
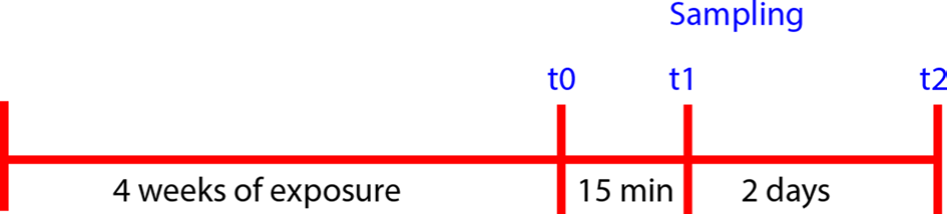
Timeline of experiment and samplings; t0: 28 days after start of experiment; t1: 15 min after the stressor (netting and 30-s air-exposure); t2: two days after first sampling.

Eggs of rainbow trout (*Oncorhynchus mykiss*) were obtained from Uckermark-Fisch GmbH (Boitzenburg, Germany) and bread at Leibniz-Institute of Freshwater Ecology and Inland Fisheries until desired size. Fingerling rainbow trout (0 + years, 24.9 ± 2 g, 13.3 ± 0.4 cm) were randomly distributed into 9 tanks (40 L, n = 30 per tank) of a flow-through system. After acclimatization, fish were exposed for 4 weeks to different concentrations (0, 5 (low), and 50 (high) mg C/L) of the FF, which were added constantly by a peristaltic pump. The throughput rate of water and FF were monitored twice per day to ensure constant exposure. Fish were fed 1.5% of their weight daily (Aller Silver, Aller Aqua, Germany) and feeding was adjusted weekly to the calculated weight gain with an assumed feed conversion ratio of 1 (13.18 g/fish in total over 28 days). During the experiment, temperature (16.5 ± 0.2 °C), pH (8.5 ± 0.1), and dissolved oxygen (8.5 ± 0.5 mg/L) were monitored daily. Nitrite (0.3 ± 0.3 mg/L), nitrate (10.4 ± 2.0 mg/L) and ammonium (< 0.02 mg/L) were measured three times a week. After taking base-line samples (t0; Fig. 7) to determine the effects of the exposure, fish were stressed by netting and 30 s of air-exposure. Fifteen minutes after stress (t1), blood was sampled to determine the effects of exposure on the immediate stress response. These fish were removed from the experiment but not sacrificed. Two days after the baseline-sampling (t2), fish were sampled again to evaluate immune and stress response after handling.

### Growth parameters

The weight and length of fish were determined at the beginning and the end of the experiment. FCR was calculated using Eq. (1); AGR was determined following Eq. (2)^58^; (n = 3 tanks). Fulton's condition factor (K) was calculated for each individual using Eq. (3) following Fulton^59^ and Barnham and Baxter^60^ .

1 $$FCR=\frac{feed \, given [\mathrm{g}]}{average \, weigth \, gained [\mathrm{g}]}$$

2 $$AGR=\frac{average \, {weigth}_{end} -\mathrm{average } \, {weigth}_{start}} \, {time} [\frac{\mathrm{g}}{\mathrm{d}}]$$

3 $$K={10}^{5}*\frac{weigth}{standard\_{length}^{3}} [\frac{\mathrm{g}}{{\mathrm{mm}}^{3}}]$$

### Sampling of blood and tissues

Four fish from each tank were collected randomly at each time point and blood was taken within less than 5 min after catching from the caudal vein. Samples were centrifuged (2000 × *g*, 5 min, 4 °C), frozen in liquid nitrogen, and stored at − 80 °C. Fish were sacrificed immediately after blood sampling and liver, spleen, visceral fat, head kidney, and gills were dissected. Liver, spleen and visceral fat were weighted to calculate hepato-somatic-index (HSI), spleen-somatic-index (SSI, Eq. 4, n = 36) and fat to body ratios (fat [g]/organs [g] and fat[mg]/total weight[g]); gills (n = 12) were shock-frozen in liquid nitrogen and stored at − 80 °C. Gill extracts were prepared by homogenization in tenfold volume (w/v) of ice-cold phosphate buffer (0.1 M, pH 7.2) and centrifuged at 10,000 × *g* for 20 min at 4 °C. The extract was used to determine immune and antioxidant parameters.

4 $$HSI or SSI=\frac{weight\left(liver \, or \, spleen\right) [\mathrm{g}]}{total \, weight [\mathrm{g}]}\times 1000$$

### Cellular response of head kidney

Head kidneys from six randomly chosen fish per group (two per tank) were aseptically removed, passed through a 70 µM cell strainer, and placed in ice-cold washing medium (RPMI 1640, 25 mM HEPES buffer, 100 U/mL Penicillin–Streptomycin, 2 mM l-glutamine (all chemicals Biowest, France), 10 U/mL heparin (Carl Roth, Germany). Erythrocytes were removed from the cell suspension by density gradient centrifugation on Histopaque 1077 (Sigma-Aldrich, USA; 500 × *g*, 45 min, 4 °C). Leucocytes were washed twice in the washing medium and adjusted to 10^7^ cells/mL in culture medium (washing medium without heparin). Cells were used to measure the respiratory burst activity by nitro blue tetrazolium (NBT) assay and to determine the phagocytic activity.

#### Nitro-blue tetrazolium (NBT) assay

The potential killing activity (reactive oxygen species (ROS) production) of head kidney cells was measured with NBT assay as described by Secombes^61^ and Chettri, et al.^62^ with minor modifications. 1*10^6^ cells were added in 9 replicates to a 96 well plate (Nunclon delta, F, clear, ThermoFisher, USA). After incubation overnight at 17 °C, non-adherent cells were removed by washing twice with 100 µL culture medium. Subsequently, cells were incubated in culture medium with 1 mg/mL NBT for 90 min at 17 °C. PMA (1 µg/mL, Sigma Aldrich), and zymosan (3.2 mg/mL, Sigma Aldrich) were used in triplicates to induce the respiratory burst. Resulting formazan was dissolved in 100 µL 2 M KOH and 100 µL DMSO and optical density was measured at 620 nm.

#### Phagocytic activity

Phagocytic activity was measured as described by Crampe, et al.^63^. 300 µL of the cell suspension were put on glass slides in duplicate. After 1 h incubation at 17 °C non-attached cells were rinsed with culture medium. Adherent cells were covered with 300 µL of heat-inactivated yeast cells (*Saccharomyces cerevisiae,* 10^8^/mL in culture medium). After 1 h of incubation at 17 °C excess yeast cells were rinsed off and slides were fixed with 100 µL methanol. Slides were stained using the Pappenheim method (Giemsa stain solution, Merck, Germany; May-Grünwald-solution, Roth, Germany)^64^. For each slide, the number of phagocytized yeast cells was enumerated for 200 phagocytes and the rate of phagocytosis (R_P_; Eq. 5) and phagocytic index (I_P_; Eq. 6) were calculated.

5 $${R}_{P}=\frac{{n}_{ingesting}}{{n}_{total}}$$

n_ingesting_ is the number of phagocytes ingesting any amount of yeast cells; n_total_ is the total number of phagocytes

6 $${I}_{P}=\frac{\sum_{i=1}^{10}C\left(i\right)\times i}{{n}_{ingested}}$$

i is the number of yeast cells ingested; C(i) number of phagocytes ingesting i yeast cells; n_ingested_ is the total number of yeast cells ingested.

### Lysozyme activity and protein content

Lysozyme activity of plasma and gill extracts was measured with the turbidimetric method^65,66^. Briefly, lyophilized *Micrococcus lysodeikticus* (Fluka, Sigma-Aldrich) (0.3 mg/mL in 0.025 M phosphate-buffer pH 6.2) was used as substrate. Reduction in absorbance at 530 nm was measured from 0 to 7 min in 1 min intervals (Infinite 200, Tecan, Switzerland). One unit of lysozyme activity was defined as the amount of enzyme causing a decrease in absorbance of 0.001/min. Protein content was measured following Bradford^67^ and used as normalization to compare different sample types. Plasma albumin content was measured after precipitation of immunoglobulin with 12% PEG (polyethylene glycol, Merck,^66^). Immunoglobulin content was considered as the difference in total protein content and albumin content.

### Reactive oxygen species (ROS) and total oxyradical scavenging capacity (TOSC)

Content of ROS and TOSC of gill extracts were measured following Amado, et al.^68^. Briefly, the conversion of H2DCF-DA (2,7-dichlorodihydrofluorescein diacetate) to the fluorescent 2,7-dichlorofluorescein by ROS was quantified at 485 nm and 528 nm for excitation and emission, respectively, at 37 °C, for 45 min in 5 min-intervals using a microplate reader (Infinite 200, Tecan). Thermally activated 2,2-azobis dihydrochloride (ABAP, 4 mM) was used to generate peroxyl radicals to measure TOSC. Using cubic spline interpolation, the area under each curve was integrated. ROS signal was normalized to protein content; TOSC was calculated following Eq. 7.

7 $$TOSC=\frac{{ROS \, Area}_{background}}{{ROS \, Area}_{ABAP}-{ROS \, Area}_{background}}$$

### Cortisol

Plasma cortisol levels were measured as duplicates using commercial ELISA kits (Cortisol Saliva ELISA, IBL) and following the manufacturer’s instructions. The absorbance was measured at 450 nm using the microplate reader Infinite 200 and the analysis software SparkControl Magellan 2.2 (Tecan Group). Plasma was diluted 1:20 at t0 and t2 and 1:50 at t1 with buffer A from the kit.

### Statistical analysis

All data are expressed as the arithmetic mean ± standard deviation (SD). The data-sets were analyzed using RStudio 1.1.453 software (https://rstudio.com/products/rstudio/download/); significant differences between exposure and control were determined by Kruskal–Wallis Test^69^ with a DunnTest (Many to one^70,71^ and BH^72^ adjustment; two-sided). Graphs are Tukey boxplots and levels of significance were expressed as p-values with p < 0.05 (*), p < 0.01 (**), and p < 0.001 (***).

### Compliance with ethical standards

Experiments were performed following the European Directive 2010/63/EU of the European Parliament and the Council of the European Union on the protection of animals used for scientific purposes and the German AnimalWelfare Act and were approved by the animal experimental ethics committee of Berlin State Office for Health and Social Affairs (LaGeSo, reference number G 0135/18).

## Supplementary Information

Supplementary Table S1.

## Data Availability

All data generated or analyzed during this study are included in this published article (and its Supplementary Information files).
