# Supplementary material for: Phenol-rich fulvic acid as a water additive enhances growth, reduces stress, and stimulates the immune system of fish in aquaculture
Source: Sci Rep. 2021 Jan 8;11:174. doi: 10.1038/s41598-020-80449-0 (PMC7794407; doi:10.1038/s41598-020-80449-0)
Supplement: Supplementary file 1 — Supplementary Table S1. [file 41598_2020_80449_MOESM1_ESM.pdf]

Phenol-rich fulvic acid as a water additive enhances growth, reduces stress, and stimulates the immune system of fish in aquaculture

Thora Lieke [a, b, *], Christian E.W. Steinberg [b, c], Bo Pan [c], Irina V. Perminova [d], Thomas Meinelt [a], Klaus Knopf [a,b], Werner Kloas [a,b]

[a] Department of Ecophysiology and Aquaculture, Leibniz-Institute of Freshwater Ecology and Inland Fisheries, Berlin, Germany

[b] Humboldt University of Berlin, Faculty of Life Sciences, Berlin, Germany

[c] Faculty of Environmental Science and Engineering, Kunming University of Science and Technology, 650500 Kunming, China

[d] Lomonosov Moscow State University, Leninskie Gory, 119991, Moscow, Russia

* Corresponding author (lieke@igb-berlin.de), Phone: +49 (0)30 64181 641, ORCID ID: 0000-0002-4345-171

Keywords: Humic substance, Innate Immunity, Persistent free radicals, Rainbow trout (*Oncorhynchus mykiss*), Stress resistance, Environment friendly therapeutant

Supplement

Table S1: Standard and reference fulvic acids from international humic substances society (IHSS) http://humic-substances.org/acidic-functional-groups-of-ihss-samples/; http://humic-substances.org/13c-nmr-estimates-of-carbon-distribution-in-ihss-samples/ and aquatic fulvic acids from rivers [74]

Fulvic Acid	Phenol content %	Aromatic content %	Reference
Suwannee River	2.91-2.84	24-22	IHSS
Elliot Soil I	nd	30	IHSS
Elliot Soil II	2.27	nd	IHSS
Pahokee Peat I	2.33	34	IHSS
Suwannee River	3.11	nd	IHSS
Pahokee Peat	1.78	nd	IHSS
Nordic Lake	3.18	31	IHSS
Missouri River FA		20.4	[74]
Yakima River FA		24.3	[74]
Ohio River FA		24.3	[74]
Minnesota groundwater FA		12.6	[74]
